# Roles of leptin on energy balance and thermoregulation in *Eothenomys miletus*

**DOI:** 10.3389/fphys.2022.1054107

**Published:** 2022-12-16

**Authors:** Huibao Chen, Hao Zhang, Ting Jia, Zhengkun Wang, Wanlong Zhu

**Affiliations:** ^1^ Key Laboratory of Ecological Adaptive Evolution and Conservation on Animals-plants in Southwest Mountain Ecosystem of Yunnan Province Higher Institutes College, School of Life Sciences, Yunnan Normal University, Kunming, China; ^2^ Yunnan College of Business Management, Kunming, China; ^3^ Engineering Research Center of Sustainable Development and Utilization of Biomass Energy Ministry of Education, Kunming, China; ^4^ Key Laboratory of Yunnan Province for Biomass Energy and Environment Biotechnology, Kunming, China

**Keywords:** *Eothenomys miletus*, leptin, thyroid hormone, energy balance, thermoregulation

## Abstract

Leptin is a hormone mainly synthesized and secreted by white adipose tissue (WAT), which regulates various physiological processes. To investigate the role of leptin in energy balance and thermoregulation in *Eothenomys miletus*, voles were randomly divided into leptin-injected and PBS-injected groups and placed at 25°C ± 1°C with a photoperiod of 12 L:12 D. They were housed under laboratory conditions for 28 days and compared in terms of body mass, food intake, water intake, core body temperature, interscapular skin temperature, resting metabolic rate (RMR), nonshivering thermogenesis (NST), liver and brown adipose tissue (BAT) thermogenic activity, and serum hormone levels. The results showed that leptin injection decreased body mass, body fat, food intake, and water intake. But it had no significant effect on carcass protein. Leptin injection increased core body temperature, interscapular skin temperature, resting metabolic rate, non-shivering thermogenesis, mitochondrial protein content and cytochrome C oxidase (COX) activity in liver and brown adipose tissue, uncoupling protein 1 (UCP1) content and thyroxin 5′-deiodinase (T_4_5′-DII) activity in brown adipose tissue significantly. Serum leptin, triiodothyronine (T_3_), thyrotropin-releasing hormone (TRH) and corticotropin-releasing hormone (CRH) concentrations were also increased significantly. Correlation analysis showed that serum leptin levels were positively correlated with core body temperature, body mass loss, uncoupling protein 1 content, thyroxin 5′-deiodinase activity, nonshivering thermogenesis, and negatively correlated with food intake; thyroxin 5′-deiodinase and triiodothyronine levels were positively correlated, suggesting that thyroxin 5′-deiodinase may play an important role in leptin-induced thermogenesis in brown adipose tissue. In conclusion, our study shows that exogenous leptin is involved in the regulation of energy metabolism and thermoregulation in *E. miletus*, and thyroid hormone may play an important role in the process of leptin regulating energy balance in *E. miletus*.

## 1 Introduction

In order to cope with seasonal changes in environmental conditions, many small mammal species, such as rodents, typically maintain energy balance by adjusting their metabolic rate, hormone, behavior and morphological changes (Klingenspor et al., 1996; Wang et al., 1999; Klingenspor et al., 2000; Bartness et al., 2002; Li and Wang, 2005; Zhao et al., 2014). And maintaining a constant body temperature is essential for their survival (Silva 2006). More and more evidence showed that leptin plays an important role in energy homeostasis of animals. Leptin is a product encoded by the obesity gene and a protein hormone with a molecular weight of 16 kD composed of 167 amino acids (Zhang et al., 1994). In humans or other mammals, leptin is mainly synthesized and secreted by adipocytes in white adipose tissue (WAT) (Considine et al., 1996; Klingenspor et al., 2000; Picó et al., 2022). It is also produced by other tissues, such as stomach, placenta, brown adipose tissue (BAT) and breast (Klingenspor et al., 1996; Hoggard et al., 1997; Bado et al., 1998; Picó et al., 2022). Leptin plays an important role in regulating animal food intake, energy expenditure and body mass (Campfield et al., 1995; Halaas et al., 1995; Pelleymounter et al., 1995; Halaas et al., 1997). Secretion of leptin is mainly regulated by body fat content, and serum leptin levels in rodent or human were positively correlated with fat storage (Considine et al., 1996; Rousseau et al., 2003; Li and Wang, 2005), therefore, leptin serves as an indicator of energy availability (Nedergaard et al., 2022). Animals that lack leptin become grossly obese, purportedly for two reasons: increased food intake (Fischer et al., 2020; Nedergaard et al., 2022) and decreased energy expenditure (Commins et al., 1999). In mice, peripheral and central injection of leptin reduced food intake and body fat (Halaas et al., 1997). It found that in seasonal small mammals such as *Microtus brandti*, *Dichotonyx groenlandicus,* and *Phodopus sungorus*, seasonal changes in food intake, body mass and body fat content have been found to be related to seasonal changes in leptin levels, which showed that leptin is involved in regulating the seasonal changes of animal body mass and energy balance (Klingenspor et al., 2000; Johnson et al., 2004; Li and Wang, 2005).

Change of energy expenditure regulated by leptin is related to the thermogenic activity of BAT. BAT is the main site of cold induced non-shivering thermogenesis (NST) in rodents. NST is dominated by the sympathetic nervous system and regulated by the hypothalamus pituitary thyroid axis (Cannon and Nedergaard, 2004; Silva 2006). Ability of NST in BAT depends on the concentration of uncoupling protein 1 (UCP1). UCP1 is a 32 kDa carrier protein uniquely expressed in the inner mitochondrial membrane of BAT, which uncouples fatty acid oxidation from adenosine triphosphate production, releasing the energy produced by metabolic fuels in the form of heat releasing energy rather than storing it as ATP (Cannon and Nedergaard, 2004), which in turn is essential for active thermoregulation in small mammals (Argyropoulos and Harper, 2002). Research showed that leptin can regulate the thermogenic capacity by increasing UCP1 content in BAT (Scarpace et al., 1997; Commins et al., 1999). Leptin also interacts with other endocrine hormones, such as melatonin, thyroid hormone, thyrotropin-releasing hormone (TRH), to jointly regulate energy homeostasis and lipid metabolism (Zimmermann Belsing et al., 2003; Hermann et al., 2006; Buonfiglio et al., 2018).

Leptin can regulate animal food intake (Campfield et al., 1995; Baicy et al., 2007; Fischer et al., 2016), and energy balance (Friedman 2019; Teixeira et al., 2021). Moreover, some studies have shown that leptin also plays an important role in thermoregulation (Luheshi et al., 1999; Fischer et al., 2016; Weiner et al., 2021). It was found that *ob/ob* mice with leptin deficiency, were not only gluttonous and obese (Halaas et al., 1995), but also had the characteristics of sub hypothermia, so *ob/ob* mice cannot survive for a long time under a low temperature environment (Joosten and van der Kroon, 1974; Trayhurn et al., 1977; Halaas et al., 1995). Later studies showed that exogenous leptin increased the body temperature in *ob/ob* mice, indicating that leptin participates in thermoregulation (Campfield et al., 1995; Halaas et al., 1995; Pelleymounter et al., 1995; Enriori et al., 2011; Kaiyala et al., 2016). Exogenous leptin injection can also increase the body temperature of *ob/ob* mice without changing the energy consumption, by opposing torpor bouts and by shifting thermoregulatory thresholds (Fischer et al., 2016; Fischer et al., 2020; Nedergaard et al., 2022). In addition, leptin therapy reduces heat loss by reducing thermal conductance (Fischer et al., 2016; Kaiyala et al., 2016).

*Eothenomys miletus* is a non-hibernating rodent, which belongs to the genus *Eothenomys* (Arvicolinae, Cricetidae, Rodentia). It is a native species of Hengduan Mountain and also endemic to China. It is distributed in some areas of Yunnan, Sichuan, Guizhou and Hubei in China, and mainly distributed in Kunming, Dali, Lijiang, Xianggelila and Ailaoshan areas in Yunnan Province. It mainly inhabits the highland mountains and forests, mostly at night, and lives in shallow surface caves with at least two kinds of grass nests in its burrows, taking fresh pulpy plants, roots and seeds of grasses as its main food. They are one of the main wild hosts and vectors of the longitudinal valley type pestis in Western Yunnan (Zhu et al., 2008). So far, there have been a number of reports on the physiological and ecological studies in *E. miletus*, such as the characteristics of thermoregulation and thermogenesis (Zhu et al., 2014), evaporative water loss (Zhu et al., 2008), changes of body mass, energy metabolism and serum leptin during cold acclimation (Zhu et al., 2010), and the effects of photoperiod on energy intake, body mass, and thermogenesis (Zhu et al., 2011). There is a positive correlation between the serum leptin concentration and body mass, and the serum leptin concentration had seasonal changes (Zhu et al., 2014). Therefore, leptin may play a role in regulating the seasonal energy balance and adaptation of *E. miletus*. However, the changes of body mass and energy metabolism of *E. miletus* after exogenous leptin injection are still unclear. In the present study, the effects of leptin on energy homeostasis and thermoregulation were studied by intraperitoneal injection of leptin in *E. miletus*. We predicted that exogenous leptin injection could enhance thermogenic capacity and reduce body mass in *E. miletus*.

## 2 Materials and methods

### 2.1 Animals

In January 2022, *E. miletus* were captured from Chenggong District, Kunming City, Yunnan Province (24°52′4″N, 102°51′57″E, 2,020 m). Kunming is located in the central Yunnan plateau, with an average altitude of 1870 m, and belongs to the mountain monsoon climate of the northern subtropical low latitude plateau. The annual average temperature is about 16.5°C, and the average minimum temperature in January is 3°C. After disinfecting and killing fleas, *E. miletus* were brought back to the laboratory of Yunnan Normal University (Chenggong Campus), and were raised in a mouse cage (260 mm × 160 mm × 150 mm), without nest material, and standard rat food (produced by Kunming Medical University) is fed. Food and water were provided *ad libitum*. The macronutrient composition of the diet was 6.2% crude fat, 20.8% crude protein, 21.5% neutral detergent fiber, 12.5% acid detergent fiber, and 10.0% ash, and the caloric value is 17.5 kJ/g. The room temperature was 25°C ± 1°C, and the light condition was 12 L: 12 D. Sixteen healthy adult individuals with similar body mass were selected and used for the experiment after 1 month of single cage adaptation.

The experiments were performed in two groups of eight animals each with a room temperature of 25°C ± 1°C. In the leptin injection group (Leptin; 4♀, 4♂), 12 L: 12 D (lights on at 8 a.m.), 0.1 ml of PBS solution containing recombinant murine leptin (ProSpec, cyt-351-c) 0.5 μg/g.d was injected intraperitoneally daily after 10 h of light; in the control group (Control; 4♀, 4♂), 0.1 ml of PBS solution was injected intraperitoneally daily at the same time as above 0.1 ml of PBS solution was used as the control treatment. The injection dose of leptin was reported by Rousseau et al. (2002). The experiment was conducted in March 2022 for 28 days (d). During the experiment, the core body temperature was measured every 2 days (measurement time, 13:00–15:00), and the body mass, food intake, water intake, RMR and NST were measured on day 0, 7, 14, 21, and 28. At 0, 14 and 28 days, the infrared thermal imager (produced by Czech Republic, WIC-640-SUW) was used to conduct thermal imaging in *E. miletus*. All animal operation procedures comply with the regulations of Animal Care and Use Committee of School of Life Sciences, Yunnan Normal University. This study was approved by the Committee (13-0901-011).

### 2.2 Determination of body temperature, body mass, food intake and water intake

The core body temperature was measured with a digital thermometer (Beijing Yezhiheng Technology Co., Ltd., XGN-1000T) (the diameter of the temperature sensor is 2 mm, and the accuracy is 0.1°C). A small amount of Vaseline was applied to the probe of the sensor, and inserts it into the rectum of the animal about 2 cm away. Record the temperature of the animal after the thermometer was stable. The interscapular skin temperature was measured by infrared thermography. The small mammal metabolic monitoring system (PRO-MRMR-8 Sable Systems International Inc, United States) was used to record body mass, food intake and water intake.

### 2.3 Metabolic rate measurement

Resting metabolic rate (RMR) and NST were measured using a small mammalian metabolic monitoring system. The gas flow rate in and out of the respiratory chamber was 200 ml/min and the size of the chamber was 32 cm × 16 cm × 15 cm. The temperature of the RMR measurement was controlled by an artificial climate chamber (Shanghai Boxun Medical Equipment Factory, Model SPX-300) (temperature fluctuation ±0.5°C), and the experimental temperature was controlled at 25.0°C ± 0.5°C within the thermoneutral zone (Zhu et al., 2008). The animals were fasted for 3 h before the measurement, and the animals were acclimatized in the respiratory chamber for 30 min, and the data were recorded every 5 min for 60 min after the animals were stabilized. At the end of the experiment, the experimental data were derived and the two lowest consecutive stable values were selected to calculate the RMR (Li and Wang, 2005).

After the RMR measurement, the animals were taken out quickly, and norepinephrine (NE) was injected subcutaneously into the interscapular to measure the maximum NST of the animals at 25°C. The amount of NE injection was 0.8 mg/kg of body weight (Zhu et al., 2010). NST was measured continuously for 60 min, data was recorded every 5 min, and two consecutive and stable highest values were selected to calculate NST (Li and Wang, 2005). In order to reduce the influence of circadian rhythm, all measurements were conducted between 9:00 and 16:00.

Thermal conductance in *E. miletus* was calculated by the following equation: C = RMR/(T_b_-T_a_), where *C* is the thermal conductance, RMR is the resting metabolic rate (ml O_2_/h), T_b_ is the body temperature (°C), and T_a_ is the ambient temperature (°C).

### 2.4 Determination of serum hormone content

At the end of the experiment, all animals were euthanized by intraperitoneal injection of pentobarbital sodium (50 mg/kg) to avoid or limit pain/distress. The blood was collected from the abdominal cavity and the collected blood was rested in a refrigerator at 4°C for 1 h, centrifuged at 4°C (4,000 rpm, 30 min), and the serum was aspirated in 2 ml centrifuge tubes, stored in a refrigerator (−80°C) and reserved. After all samples were collected, the contents of leptin, triiodothyronine (T_3_), thyroxine (T_4_), TRH and corticotropin-releasing hormone (CRH) in serum are determined by mouse enzyme linked immunosorbent assay (ELISA). Leptin assay kit (Product No. YX-E20015M), T_3_ assay kit (Product No. YX-E20366M), T_4_ assay kit (Product No. YX-E20363M), TRH assay kit (Product No. YX-E20509M) and CRH assay kit (Product No. YX-E22127M) were purchased from Preferred Biotechnology Co. (Shanghai, China). The inter- and intra-assay variations were 7.6% and 3.3%, respectively, for leptin; 7.2% and 3.2% for T3; 7.8% and 3.4% for T4; 8.2% and 3.6% for TRH, and 8.7% and 3.2% for TRH.

### 2.5 Determination of protein content and enzyme activity in liver and BAT

Quickly dissect the animals, carefully separate the liver, interscapular BAT, weigh them (to the nearest 0.001 g), put them into 5 ml and 2 ml centrifuge tubes respectively, place them in liquid nitrogen, and then transfer them to a low-temperature refrigerator (− 80°C) for storage and standby. After all samples were collected, mitochondria in liver and BAT was extracted with Tissue Mitochondria Isolation Kit (Shanghai Biyuntian Biotechnology Co., Ltd., Product No. C3606). The content of mitochondrial protein (MP) in liver and BAT was determined with BCA Protein Assay Kit (Product No. YX-E20015M). UCP1 content, cytochrome C oxidase (COX, complex IV) activity, T_4_5′-deiodinase (T_4_5′-DII) activity in BAT and COX activity in liver were determined by mouse ELISA kits. UCP1 assay kit (Product No. YX-E22121M), COX assay kit (Product No. YX-E22118M), T_4_5′-DII assay kit (Product No. YX-E22129M) were purchased from Shanghai Preferred Biotechnology Co. (Shanghai, China). The experimental operation was carried out according to the instructions. The inter- and intra-assay variations were 5.6% and 3.1%, respectively, for UCP1; 5.0% and 2.6% for COX, and 5.3% and 3.2% for T45′-DII.

### 2.6 Measurement of body composition and digestive organs

Gastrointestinal tract (stomach, small intestine, colon, and cecum) were extracted firstly, weighed with content (to 1 mg) and measured with a ruler (to 0.1 cm), respectively. Then, the contents of the gastrointestinal tract were removed and weighed to get wet mass (to 1 mg). Finally, heart, liver, lung, gonad, spleen and kidneys were removed respectively. All tissues and the remaining carcass were weighed (to 1 mg and 0.01 g, respectively) to get wet mass, dried in an oven at 60 C for at least a week and then reweighed to get the dry mass. The carcass fat and protein were measured after the carcass was dried to constant weight. See the literature for the detailed determination method (Ferreira et al., 2007).

### 2.7 Statistical analysis

Data were analyzed using SPSS 22.0 software (SPSS Inc., Chicago, IL, United States). Before all statistical analyses, data were examined for normality and homogeneity of variance using Kolmogorov-Smirnov and Levene tests, respectively. Differences in physiological indicators between the different sexes of *E. miletus* were not significant, so all data were combined and counted. Continuous changes in body mass, body temperature, food intake, water intake, RMR and NST were detected by repeated-measures ANOVA. Differences in body mass between groups were analyzed by independent samples *t*-test. One-way ANOVA was used for between-group differences in body core temperature, interscapular skin temperature, and thermal conductance. Group differences in food intake, water intake, RMR, NST, serum hormone content, protein content in liver and BAT, and enzyme activity were analyzed by a one-way analysis of covariance (ANCOVA) with body mass as a covariate. Pearson correlation analysis was performed to determine the correlations between serum leptin and body core temperature, body mass loss, food intake, NST, RMR, and UCP1 content, serum T_3_ and T_4_5′II activity, and the correlation between serum T_3_ and T_4_5′-DII activity and NST, and the correlation between NST and interscapular BAT temperature. Data were expressed as means ± standard error (SE), and *p* < .05 was considered to be statistically significant.

## 3 Results

### 3.1 Body temperature

**FIGURE 1:**
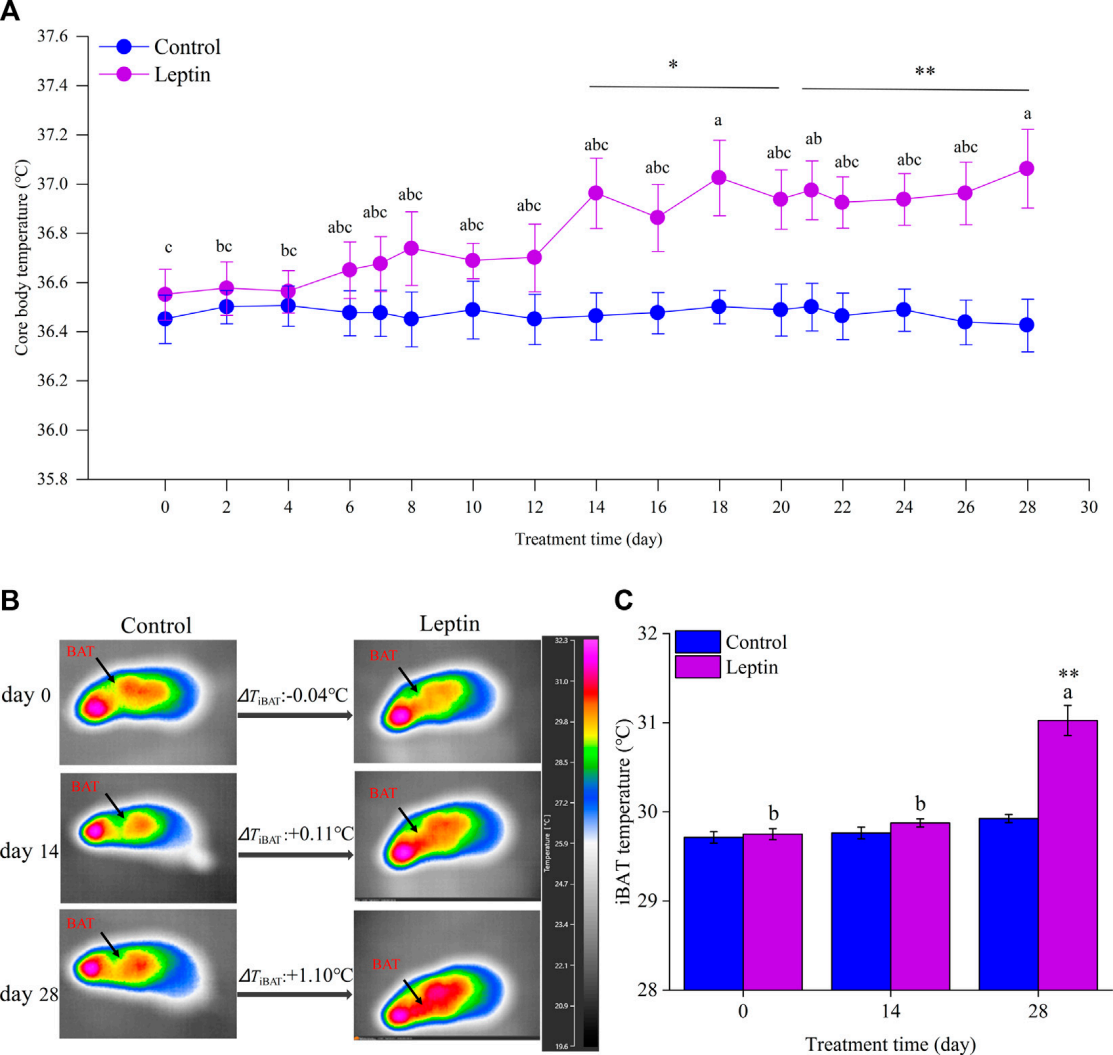
Effect of leptin on core body temperature **(A)** and interscapular skin temperature **(B,C)** in *Eothenomys miletus.* Different letters (a–c) indicate significant differences within the leptin group (*p* < .05); *:*p* < .05, **:*p* < .01 indicates a significant difference between the leptin and control groups.

Before leptin injection, there was no difference in body core temperature between the control group and leptin group (*F*_1,14_ = 0.491, *p* = 0.495). With the increase of domestication time, there was no significant change in body core temperature in the control group (*F*_14,98_ = 0.077, *p* = 0.994), but in leptin group it showed significant changes (*F*_14,98_ = 9.181, *p* = 0.019). From the 14th day to the end of the experiment, the body core temperature of the animals in the two groups had significant differences (14 days: *F*_1,14_ = 8.446, *p* = 0.011, 28 days: *F*_1,14_ = 10.975, *p* = 0.005, Figure 1A). On the 28th day, the body core temperature of the control group and leptin group was (36.4 ± 0.1)°C and (37.1 ± 0.2)°C, respectively, and the leptin group was 0.6°C higher than the control group. Analysis using thermography showed that there was no difference in interscapular skin temperature between the two groups before the experiment (*F*_1,14_ = 0.175, *p* = 0.682), and at 28 days the difference in interscapular skin temperature between the two groups was statistically significant (*F*_1,14_ = 39.626, *p* < 0.001), and the leptin group was 1.1°C higher than the control group (*F*_2,14_ = 43.109, *p* < 0.001), and there was no significant difference in interscapular skin temperature during the experiment in the control group in *E. miletus* (*F*_2,14_ = 4.036, *p* = 0.067, Figures 1B,C).

### 3.2 Body mass, food intake and water intake

**FIGURE 2:**
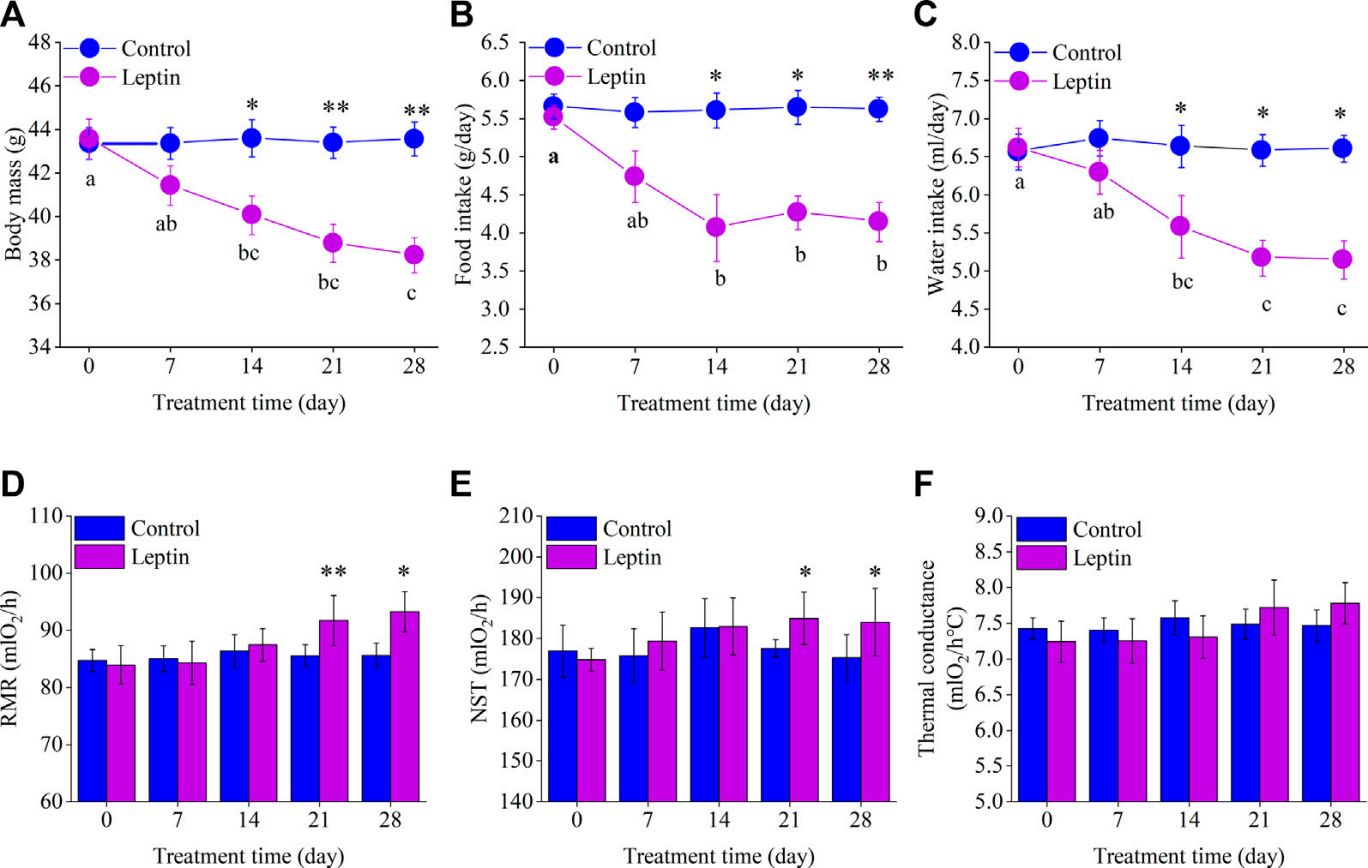
Effect of leptin on body mass **(A)**, food intake **(B)**, water intake **(C)**, RMR **(D)**, NST **(E)** and thermal conductance **(F)** in *Eothenomys miletus.* Different letters (a–c) indicate significant differences (*p* < .05) within the leptin group; *:*p* < .05, **:*p* < .01 indicates a significant difference between the leptin and control groups.

Before the experiment, body mass in control group was 43.4 ± 0.7 g, and that of the leptin group was 43.6 ± 0.9 g, there was no significant difference between the two groups (*t* = −1.77, *df* = 14, *p* = 0.862). From the 14th day on and after, there was a significant difference between the body mass of the control group and that of the leptin group. On day 14, the body mass of leptin group was significantly lower than that of the control group (*t* = 2.866, *df* = 14, *p* = 0.012), which was 8.075% lower than that of the control group. On day 28, the body mass of the control and leptin groups were 43.6 ± 0.8 g and 38.2 ± 0.8 g respectively, with a significant difference between the two groups (*t* = 4.755, *df* = 14, *p* = 0.000), and the leptin group was 12.3% lower than the control group. There was no significant change in the body mass of the control group (*F*_4,28_ = 0.123, *p* = 0.877); while the leptin group showed significant differences, showing a significant decrease (*F*_4,28_ = 43.038, *p* = 0.000), body mass on the 28th day was significantly lower than that on the 0th day, 12.3% lower than that on the 0th day (Figure 2A).

There was no significant difference in food intake between the control group and leptin group before the experiment (*F*_1,13_ = 0.292, *p* = 0.598). On day 14–28, food intake in the leptin group was significantly lower than that of 27.5%, 24.5%, and 26.3% compared with the control group (day 14: *F*_1, 13_ = 5.571, *p* = 0.035; day 21: *F*_1, 13_ = 5.440, *p* = 0.036; day 28: *F*_1, 13_ = 14.918, *p* = 0.002), and food intake in the leptin group were significantly reduced (*F*_4, 28_ = 4.130, *p* = 0.027). Among them, the intake on the 28th day can be 25% lower than the first day; differences in food intake was not significant in the control group (*F*_4, 28_ = 0.029, *p* = 0.962, Figure 2B).

Before the experiment, the water intake of the control and leptin groups of *E. miletus* was 6.6 ± 0.2 ml and 6.6 ± 0.3 ml, respectively, which were not significantly different (*F*_1,13_ = 0.031, *p* = 0.862). After day 14, water intake was significantly lower in the leptin group than in the control group. On day 14, the water intake of the leptin group was 15.8% lower than that of the control group (*F*_1,13_ = 6.402, *p* = 0.025). At the end of the experiment (day 28), the water intake of the leptin group was 22.1% lower than that of the control group (*F*_1,13_ = 6.955, *p* = 0.021). Water intake in the leptin group was significant and showed a significant decrease (*F*_4,28_ = 5.352, *p* = 0.017), with 22.2% lower water intake at d 28 compared to d 0, while variations of water intake in the control group was not significant (*F*_4,28_ = 0.109, *p* = 0.866, Figure 2C).

### 3.3 Metabolic rate

Before the experiment, there was no difference in RMR and NST between the control group and leptin group (RMR: *F*_1,13_ = 0.123, *p* = 0.732; NST: *F*_1,13_ = 0.256, *p* = 0.622). At 21 days, RMR and NST of the two groups were significantly different (RMR: *F*_1,13_ = 10.594, *p* = 0.006; NST: *F*_1,13_ = 5.711, *p* = 0.033). RMR and NST of leptin group were 7.2% and 4.2% higher than those of the control group respectively; At 28 days, there was a significant difference in RMR (RMR: *F*_1,13_ = 7.416, *p* = 0.017) and NST (NST: *F*_1,13_ = 4.971, *p* = 0.044) between the two groups. RMR and NST in leptin group increased by 9.0% and 5.0% respectively compared with the control group (Figures 2D, E). With the prolongation of leptin injection time, there was no significant change in RMR and NST in the control group (RMR: *F*_4,28_ = 0.128, *p* = 0.971; NST: *F*_4,28_ = 0.412, *p* = 0.799); The RMR and NST of leptin group animals also had no significant change with the prolongation of leptin injection time (RMR: *F*_4,28_ = 1.769, *p* = 0.163; NST: *F*_4,28_ = 0.466, *p* = 0.617; Figures 2D, E).

Leptin injection had no significant effect on the thermal conductance of the *E. miletus* (d 0, *F*_1,14_ = 0.314, *p* = 0.584; d 7, *F*_1,14_ = 0.169, *p* = 0.687; d 14, *F*_1,14_ = 0.490, *p* = 0.495; d 21, *F*_1,14_ = 0.279, *p* = 0.606; d 28, *F*_1,14_ = 0.732, *p* = 0.407; Figure 2F).

### 3.4 Protein content and enzyme activity in liver and BAT

**FIGURE 3:**
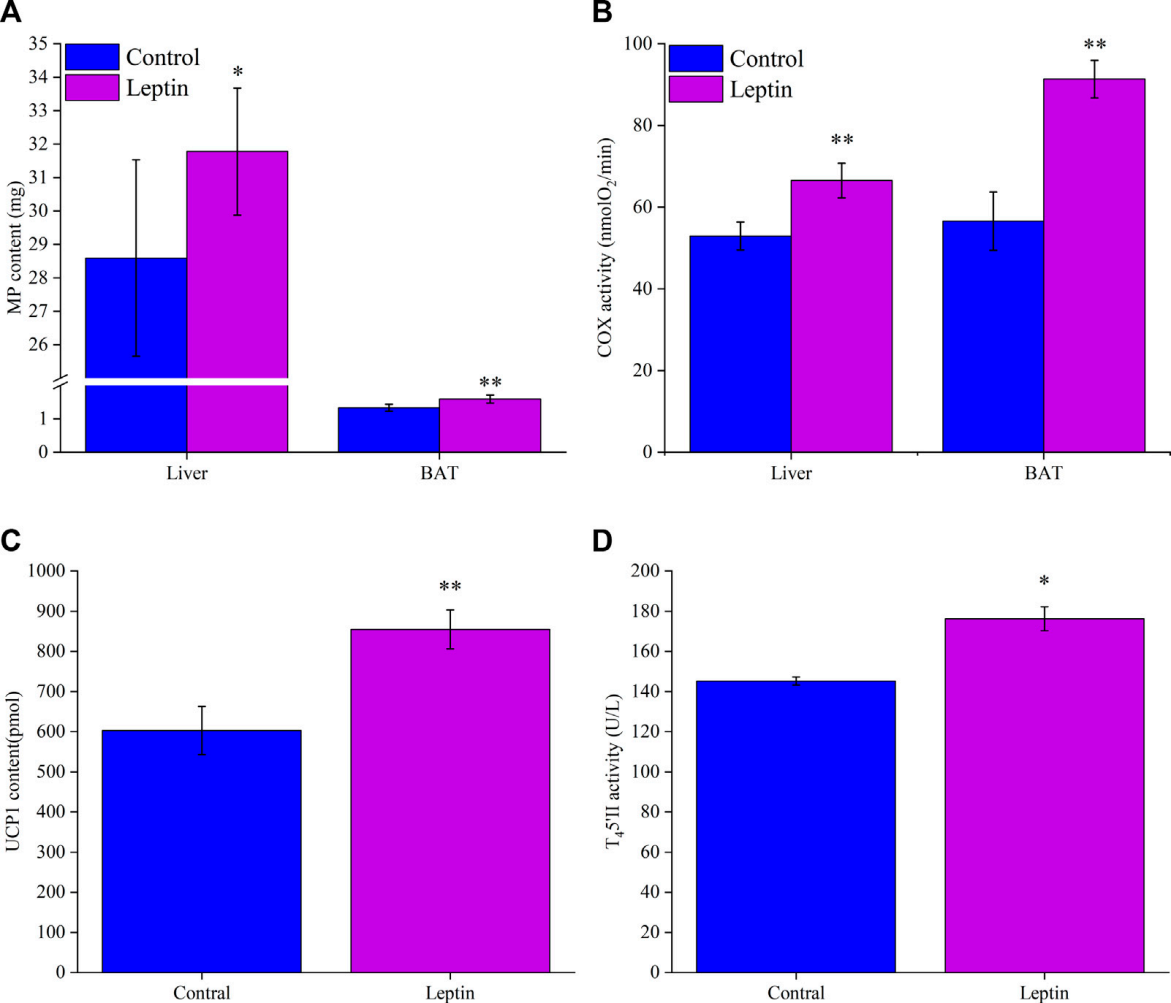
Leptin affects mitochondrial protein content **(A)** and COX activity **(B)** of *Eothenomys miletus* in liver and BAT, UCP1 content **(C)** and T_4_5′-DII activity **(D)** in BAT. *:*p* < .05, **:*p* < .01 indicates a significant difference between the leptin and control groups.

After 28 days of leptin injection, the content of MP in liver and BAT was significantly higher than that in the control group (liver: *F*_1,13_ = 4.735, *p* = 0.049; BAT: *F*_1,13_ = 18.568, *p* = 0.001; Figure 3A). COX activity in liver and BAT was significantly higher than that in control group (liver: *F*_1,13_ = 14.966, *p* = 0.002; BAT: *F*_1,13_ = 12.107, *p* = 0.004; Figure 3B). After leptin injection, the content of UCP1 in leptin group was significantly higher than that in control group (*F*_1,13_ = 25.955, *p* < 0.001; Figure 3C). Leptin injection significantly increased T_4_5′-DII activity in BAT (*F*_1,13_ = 4.724, *p* = 0.049; Figure 3D), which was 21.4% higher than that in control group.

### 3.5 Serum hormone content

**FIGURE 4:**
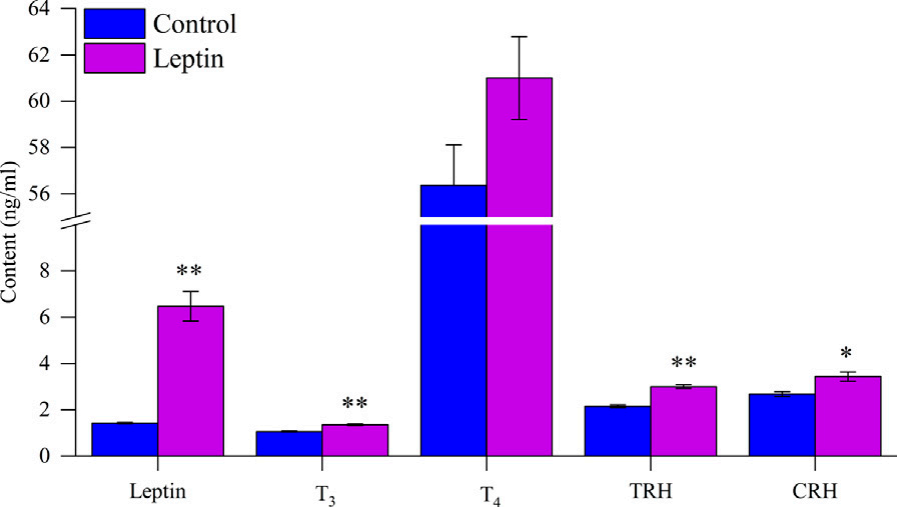
Effect of leptin on serum hormone levels in *Eothenomys miletus*. *:*p* < .05, **:*p* < .01 indicates a significant difference between the leptin and control groups.

**FIGURE 5:**
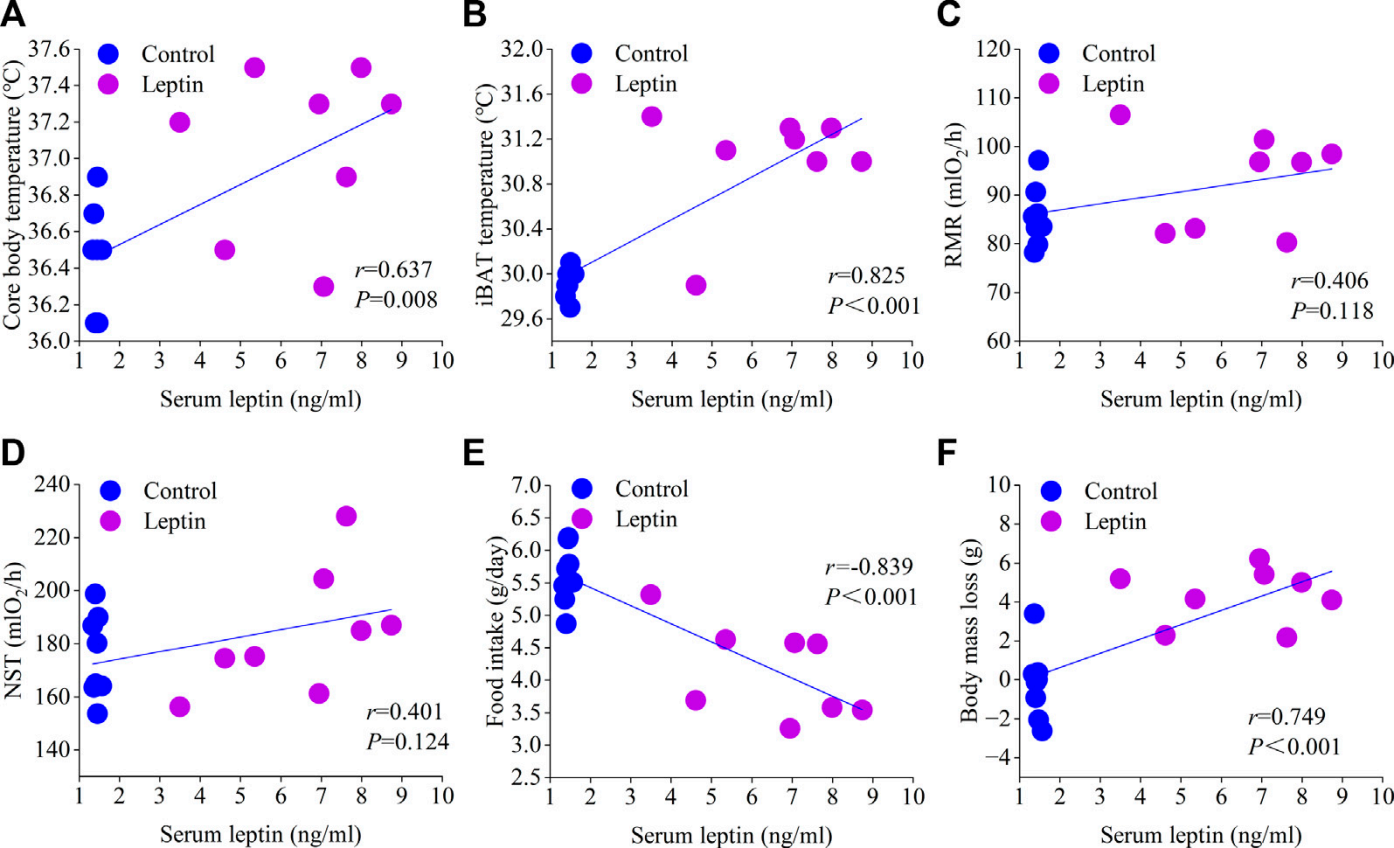
Correlation of serum leptin with core body temperature **(A)**, interscapular skin temperature **(B)**, RMR **(C)**, NST **(D)**, food intake **(E)** and body mass loss **(F)**.

**FIGURE 6:**
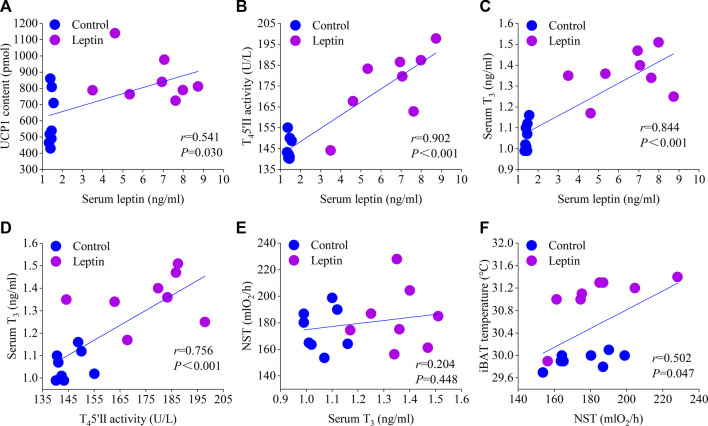
Correlation of serum leptin with UCP content **(A)**, T_4_5′-DII activity **(B)**, and T_3_ content **(C)**; correlation of serum T_3_ with T_4_5′-DII activity **(D)** and NST **(E)**; correlation of NST with interscapular skin temperature **(F)**.

After leptin injection, serum leptin level increased significantly (*F*_1,13_ = 14.831, *p* = 0.002), T_3_ increased significantly (*F*_1,13_ = 12.054, *p* = 0.004), T_4_ content did not change significantly (*F*_1,13_ = 4.231, *p* = 0.060), TRH content was significantly higher than the control group (*F*_1,13_ = 23.015, *p* < 0.001), and CRH content was significantly higher than the control group (*F*_1,13_ = 6.714, *p* = 0.022; Figure 4). Correlation analysis showed that serum leptin content was positively correlated with core body temperature, interscapular skin temperature, RMR, UCP1, NST, T_4_5′-DII activity, serum T_3_ content, body mass loss, and negatively correlated with food intake (Figures 5, 6); T_4_5′-DII activity and NST were positively correlated with serumT_3_ content (Figure 6); NST was positively correlated with interscapular skin temperature (Figure 6).

### 3.6 Body composition and digestive tract morphology

After 4 weeks of leptin injection, there were significant differences in several measured parameters between the control and leptin groups of *E. miletus* (Table 1). Leptin injection significantly reduced the carcass fat, carcass wet mass, subcutaneous WAT, perisexual WAT, and mesenteric WAT of *E. miletus* compared with the control group, and the other body composition was not significantly affected by leptin (Table 1). After leptin injection, the mass with content of stomach, cecum wet mass, and mass with content of large intestine of the leptin group were significantly lower than those of the control group, and the rest of the digestive tract morphology was not significantly affected by leptin (Table 2).

**TABLE 1:** Effects of leptin on body composition in *Eothenomys miletus*.

Body compositions	Control	Leptin	*F*	*P*
Sample size	8 (4♀, 4♂)	8 (4♀, 4♂)		
Body length (cm)	11.025 ± 0.186	10.850 ± 0.216	0.028	0.870
Tail length (cm)	4.225 ± 0.122	4.137 ± 0.122	0.233	0.638
Liver wet mass (g)	1.642 ± 0.139	1.517 ± 0.082	1.686	0.217
BAT wet mass (g)	0.217 ± 0.016	0.211 ± 0.012	4.066	0.052
Interscapular WAT mass (g)	0.108 ± 0.010	0.104 ± 0.010	0.411	0.533
Subcutaneous WAT mass (g)	2.382 ± 0.106	1.584 ± 0.142	11.002	0.005
Perigonadal WAT mass (g)	0.663 ± 0.031	0.394 ± 0.022	14.878	0.002
Retroperitoneal WAT mass (g)	0.190 ± 0.008	0.143 ± 0.013	1.753	0.208
Mesenteric WAT mass (g)	0.389 ± 0.015	0.273 ± 0.014	5.696	0.033
Carcass wet mass (g)	29.064 ± 0.302	26.318 ± 0.320	6.321	0.026
Carcass dry mass (g)	15.789 ± 0.238	13.118 ± 0.398	5.504	0.035
Carcass fat (g)	7.28 ± 0.106	5.028 ± 0.101	87.840	<0.001
Carcass protein (g)	6.477 ± 0.211	6.281 ± 0.321	0.191	0.669
Heart wet mass (g)	0.2286 ± 0.010	0.214 ± 0.010	0.006	0.941
Heart dry mass (g)	0.060 ± 0.002	0.055 ± 0.003	0.124	0.730
Spleen wet mass (g)	0.077 ± 0.006	0.068 ± 0.005	0.008	0.932
Spleen dry mass (g)	0.022 ± 0.001	0.020 ± 0.001	0.371	0.553
Lung wet mass (g)	0.249 ± 0.014	0.256 ± 0.015	1.508	0.241
Lung dry mass (g)	0.060 ± 0.004	0.065 ± 0.006	2.601	0.131
Kidneys wet mass (g)	0.364 ± 0.026	0.349 ± 0.020	0.125	0.729
Kidneys dry mass (g)	0.113 ± 0.011	0.099 ± 0.005	0.559	0.468
Testis wet mass(g)	0.633 ± 0.056	0.568 ± 0.106	2.192	0.199
Testis dry mass (g)	0.112 ± 0.011	0.110 ± 0.015	1.541	0.270

Data are mean ± SE. Values are significantly different at *p* < .05, determined by One-Way ANOVA, with body mass as a covariate.

**TABLE 2:** Effects of leptin on the morphology of digestive tract in *Eothenomys miletus.*

Digestive tract morphology	Control	Leptin	*F*	*P*
Stomach				
Length (cm)	1.725 ± 0.045	1.613 ± 0.064	1.401	0.258
Mass with content (g)	0.983 ± 0.024	0.750 ± 0.055	5.033	0.043
Wet mass (g)	0.287 ± 0.017	0.249 ± 0.026	1.387	0.260
Dry mass (g)	0.078 ± 0.004	0.072 ± 0.003	0.081	0.780
Small intestine				
Length (cm)	31.206 ± 1.571	30.488 ± 1.748	0.938	0.351
Mass with content (g)	1.538 ± 0.181	1.598 ± 0.132	0.233	0.637
Wet mass (g)	0.736 ± 0.083	0.524 ± 0.061	0.018	0.897
Dry mass (g)	0.123 ± 0.015	0.087 ± 0.014	0.014	0.908
Caecum				
Length (cm)	8.725 ± 0.327	9.088 ± 0.406	2.474	0.140
Mass with content (g)	2.131 ± 0.169	2.409 ± 0.436	0.544	0.474
Wet mass (g)	0.483 ± 0.035	0.323 ± 0.020	5.931	0.030
Dry mass (g)	0.086 ± 0.010	0.072 ± 0.009	0.108	0.747
Large intestine				
Length (cm)	17.173 ± 0.986	16.225 ± 0.880	0.012	0.913
Mass with content (g)	1.226 ± 0.109	0.731 ± 0.091	5.642	0.034
Wet mass (g)	0.347 ± 0.040	0 302 ± 0.047	0.001	0.972
Dry mass (g)	0.061 ± 0.006	0.059 ± 0.006	0.017	0.897

Data are mean ± SE. Values are significantly different at *p* < .05, determined by One-Way ANOVA, with body mass as a covariate.

## 4 Discussion

Thermoregulation is one of the most important mechanisms in animal physiology, which is essential not only to prevent cellular damage from physiological temperature extremes, but also to optimize biological activity and body function (Rezai-Zadeh and Münzberg, 2013). Studies showed that leptin plays a key role not only in energy balance, but also in thermoregulation (Rezai-Zadeh and Münzberg, 2013), and most studies have shown that higher leptin levels increased body temperature in animals (Enriori et al., 2011; Dodd et al., 2014; Rezai- Zadeh et al., 2014). In the present study, leptin injection for 28 days caused a significant increase in core body temperature and interscapular skin temperature. Consistently, leptin injection similarly increased core body temperature and interscapular skin temperature in rats (de Git et al., 2019), and obese mice (Enriori et al., 2011). Whereas in leptin-deficient *ob/ob* mice usually exhibit a subhypothermic profile at room temperature and they become extremely hypothermic when directly exposed to cold (Trayhurn et al., 1977), the body temperature of *ob/ob* mice increased after long-term leptin treatment (Pelleymounter et al., 1995; Kaiyala et al., 2016). Lower body temperature and energy expenditure were also found in patients with congenital leptin deficiency, whereas the introduction of exogenous leptin normalized body temperature and energy expenditure (Farooqi et al., 1999), and these results suggested that leptin is directly involved in thermoregulation.

Leptin induces thermogenesis and thermoregulation partly through its effect on thyroid hormone axis (Ukropec et al., 2006). Thyroid hormone is also an important hormone to regulate thermogenesis and body temperature. Severe deficiency of thyroid hormone leads to hypothermia (Silva, 2006). Leptin deficiency is not only related to hypothermia (Trayhurn et al., 1977), but also to the reduction of circulating thyroid hormone levels (Deem et al., 2018), and the above two conditions can be improved by leptin treatment (Pelleymounter et al., 1995; Kaiyala et al., 2016; Deem et al., 2018). T_4_5′-DII is expressed in hypothalamus, WAT, BAT and skeletal muscle (Bianco et al., 2002), which is necessary for adaptive thermogenesis (Mullur et al., 2014). T_4_5′-DII can locally activate T_4_ to become active T_3_, which is the key mechanism of thyroid hormone regulating metabolism (Mullur et al., 2014). Moreover, the study suggests that the high level of serum T_3_ may be the reason for the increase of deiodinase activity in animals taking leptin for a long time (Cettour-Rose et al., 2002). Leptin can up regulate the activity of thyroid deiodinase (Cettour-Rose et al., 2002; Lisboa et al., 2003). Studies have confirmed that leptin administered to the central nervous system for 3 days can increase the activity of T_4_5′-DII in rat BAT (Cettour-Rose et al., 2002). More importantly, the 30-min thermal response of the interscapular BAT of T_4_5′-DII gene knockout mice to NE infusion is reduced, which clearly shows that the ability of T_4_5′-DII gene knockout mice to generate heat from BAT is reduced (de Jesus et al., 2001), the administration of T_4_5′-DII inhibitor led to the decrease of UCP-1 expression in BAT (Branco et al., 1999). These results indicated that T_4_5′-DII is a key regulator of function and UCP1 induction in BAT, and is necessary for the normal development of BAT and the differentiation of BAT cells. In our study, the serum T_3_ content of leptin group was significantly higher than that of the control group, leptin injection also enhanced the activity of T_4_5′-DII in BAT. The correlation analysis showed that the serum leptin content was positively correlated with T_4_5′-DII activity and T_3_ content, T_4_5′-DII activity was positively correlated with T_3_ content. Therefore, leptin may increase the concentration of T_3_ by increasing T_4_5′-DII activity in BAT, so as to increase thermogenesis and body temperature, suggesting that thyroid hormone may be one of the important mediators of leptin on thermogenesis and thermoregulation in *E. miletus* (Deem et al., 2018). Moreover, TRH and CRH also participate in the regulation of animal energy balance. Studies showed that intracerebroventricular injection of CRH increased the interscapular BAT temperature and rectal temperature of Syrian hamsters (Shintani et al., 2005). In the present study, the contents of TRH and CRH in the serum increased significantly, suggesting that leptin may interact with TRH and CRH during thermoregulation.

Leptin may also regulate body temperature by regulating thermogenesis. Some researchers believe that the hypothermia of *ob/ob* mice is due to the lack of leptin, which makes them unable to reduce the thermal conductance (Kaiyala et al., 2016). Previous research data on *ob/ob* mice showed that leptin reduced its thermal conductance at low ambient temperature (14°C) and high ambient temperature (30°C), but had no effect on the thermal conductance of *ob/ob* mice at room temperature (22°C). Similarly, in the present study, leptin had no significant effect on the thermal conductance of *E. miletus* at room temperature. However, in the current study, the effect of leptin on the thermal conductance of *E. miletus* at low ambient temperature and high ambient temperature was not evaluated, which needs further study. Furthermore, some studies have shown that leptin does not increase thermogenesis, but increases defensive body temperature by reducing heat loss in the tail (Fischer et al., 2016). Therefore, whether leptin can regulate the temperature of rats by tail heat loss is also worthy of attention, because tail heat loss (controlled by the vasodilation and contraction of tail blood vessels) plays an important role in the temperature regulation of rodents, although in this study, leptin can enhance the thermogenesis capacity of *E. miletus*, which may be the main reason for increasing the temperature.

Enhanced thermogenesis in many small mammals has been found to be associated with increased MP concentrations, COX activity, UCP1 mRNA levels and protein expression (Wang et al., 1999; Li and Wang, 2005; Wang et al., 2006). Similarly, the results of the present study showed that MP concentration, and COX activity increased in the liver and BAT of the leptin group, suggesting that the total respiratory capacity of the liver and BAT was increased after leptin injection, which was consistent with the leptin group having higher RMR and NST. Leptin-regulated changes in energy expenditure are associated with thermogenic activity in BAT, and UCP1-mediated NST in BAT plays an important role in maintaining constant body temperature in many mammals (Tiraby et al., 2003). Studies have demonstrated that high leptin concentrations can also induce adaptive thermogenesis by increasing UCP1 expression in BAT (Pelleymounter et al., 1995; Levin et al., 1996; Commins et al., 1999; Scarpace et al., 1997; Johnson et al., 2004), thereby increasing energy expenditure (Scarpace et al., 1997; Commins et al., 1999). Leptin deficiency leads to reduced UCP1 expression and reduced thermogenic capacity in animals, and supplementation with exogenous leptin can correct this deficiency (Commins et al., 1999). The results of the present study were consistent with the conclusion that leptin increases the thermogenic activity of BAT. Compared with the control group, leptin injection significantly increased the content of UCP1 in BAT and enhanced the NST and increased the interscapular skin temperature in the *E. miletus*. After correlation analysis, it was shown that interscapular skin temperature and NST were positively correlated (Figure 6F), suggesting that the increased interscapular skin temperature may mainly reflect the increase in thermogenesis. The present study and most other studies suggest that leptin increases energy expenditure by increasing thermogenesis in the BAT, including increased expression of UCP1 or UCP1 mRNA (Scarpace et al., 1997; Commins et al., 1999). However, contrary experimental results also exist, such as the administration of leptin to cold-adapted rats decreases food intake and BAT thermogenesis (Abelenda et al., 2003), and obese F344 × BN aged rats show diminished anorexic and febrile responses to both peripheral and central leptin treatment (Shek and Scarpace, 2000). Other studies have shown that leptin is not a thermogenic hormone, and it can increase animal body temperature without changing thermogenesis. It is possible to increase body temperature without increasing heat thermogenesis, because mice can reduce heat loss through tail vasoconstriction (Fischer et al., 2016; Kaiyala et al., 2016). Nedergaard et al. (2022) reviewed available data and concluded that most papers implying a thermogenic effect of leptin have based this on misconstrued division by body mass, and they have collected evidence that the remaining observations that imply that leptin is a thermogenic hormone are better understood as implying that leptin is an anti-torpor hormone. The information on body energy reserves is normally conveyed by leptin but since the *ob/ob* mice obviously lack this information, they react as if they are without energy reserves and therefore readily enter torpor.

Research showed that serum leptin plays an important role in controlling body mass and body fat content by reducing food intake and increasing energy expenditure (Halaas et al., 1995; Levin et al., 1996; Klingenspor et al., 2000; Johnson et al., 2004). In the present study, leptin injection resulted in a significant reduction in body mass and food intake in *E. miletus*, which is consistent with results observed in other animals under leptin injection, including *Lasiopodomys brandtii* (Tang et al., 2009), *Phodopus sungorus* (Klingenspor et al., 2000), *ob/ob* and wild type mice (Halaas et al., 1995; Pelleymounter et al., 1995; Levin et al., 1996), Sprague Dawley rat (Gullicksen et al., 2002) *Microtus agrastis* (Król et al., 2006), and *Spermophilus dauricus* (Xing et al., 2016). Consistent with the body mass loss, leptin injection also significantly reduced the body fat content of *E. miletus*, indicating that compared with the control group, the energy consumption and fat carcass reserve consumption of the leptin group *E. miletus* increased. At present, there is little information about the effect of leptin on the synthesis or turnover of muscle protein *in vivo*, but some studies have shown that leptin inhibits protein decomposition in cultured C_2_C_12_ myoblasts (Ramsay 2003), but our research showed that leptin has no significant effect on carcass protein. The results of this study showed that the serum leptin concentration was significantly increased in the leptin group, and the body mass and food intake were reduced in the leptin group. The correlation analysis showed that the serum leptin level was positively correlated with body mass loss and negatively correlated with food intake, indicating that increasing of the leptin concentration suppressed the food intake of *E. miletus* and thus reduced the body mass. Leptin injection also significantly reduced the stomach and large intestine contents mass of *E. miletus*, which may be due to the suppression of feeding and water intake by leptin.

## 5 Conclusion

In conclusion, the results of the present study suggested that exogenous leptin reduces food intake, carcass fat and body mass, enhanced thermogenesis and increased body temperature, and that leptin was involved in energy balance and thermoregulation in *E. miletus*. Moreover, thyroid hormone may be an important mediator of leptin regulation of energy balance and thermoregulation in *E. miletus.*

## Data Availability

The raw data supporting the conclusion of this article will be made available by the authors, without undue reservation.

## Abbreviations

RMR, Resting metabolic rate; NST, Nonshivering thermogenesis; BAT, Brown adipose tissue; COX, Cytochrome C oxidase; T_4_5′-DII, Thyroxin 5′-deiodinase; UCP 1, Uncoupling protein 1; T_3,_ Triiodothyronine; T_4,_ Thyroxine; TRH, Thyrotropin-releasing hormone; CRH, Corticotropin-releasing hormone.
